# “Y” anastomosis, a solution in doubtful venous anastomosis: a case report and review of the literature

**DOI:** 10.1186/s13256-023-04177-5

**Published:** 2023-12-02

**Authors:** Soha Mohammadi, Nima Taghizadeh Mortezaei, Arash Abdollahi, Shabahang Mohammadi

**Affiliations:** 1 ENT and Head & Neck Research Center, The Five Senses Health Institute, School of Medicine, Iran University of Medical Sciences, Tehran, Iran; 2 School of Medicine, Iran University of Medical Sciences (IUMS), Tehran, Iran; 3 Student Research Committee, Iran University of Medical Sciences, Tehran, Iran; 4 Department of Otolaryngology-Head and Neck Surgery, Firoozgar Hospital, Iran University of Medical Sciences, Valadi Street, Valiasr Sq, Tehran, Iran

**Keywords:** Y anastomosis, End-to-end anastomosis, Microsurgery, Squamous cell carcinoma of head and neck, Free tissue flaps

## Abstract

**Background:**

Lower lip squamous cell carcinoma is a significant subtype of head and neck cancer, constituting about 25–30% of cases. Traditional surgical methods, like primary closure, have limitations in managing large resections of lip tumors. Recent advancements in surgical techniques, particularly free flaps, have shown promising results in addressing these challenges. The Y-shaped anastomosis is an innovative approach aimed at enhancing the efficiency of microvascular free flap surgeries for improved lip cancer reconstruction outcomes.

**Case presentation:**

A 77-year-old Persian male with lower lip squamous cell carcinoma underwent tumor resection with a 2 cm safety margin, bilateral neck dissection, and lip reconstruction using the right radial forearm free flap. The surgery incorporated a Y-shaped anastomosis to improve venous pedicle outcomes.

**Conclusion:**

In this case, it was decided not to open the first anastomosis but to add the second end to the side one to provide two vascular supports for the venous anastomosis. Y anastomosis makes the surgery easier and decreases complications resulting from vascular size mismatch.

## Introduction

Head and neck cancer is the world’s sixth most common type of cancer [1]. Among head and neck cancers, oral cancer is a prevalent form, with lower lip squamous cell carcinoma (SCC) accounting for over 25–30% of cases [1, 2]. Lip SCCs risk factors include male sex, older age, fair skin, prolonged sun or ultraviolet (UV) exposure in occupations like farming and fishing, tobacco and alcohol consumption, and viral factors, such as human papillomavirus (HPV) 16 and 24, and herpes virus (HSV) 1 and 2 [2, 3]. Despite these risks, LSCCs generally have a high survival rate [4].

Over the past two decades, advancements in technology, surgical techniques, and anatomical understanding have improved the management of head and neck cancers [5]. Consequently, the surgical approach for lip cancer has also seen notable progress, with decisions largely dependent on the tumor’s size and location [6].

There are different methods for lip cancer reconstruction, including primary closure, and free flaps [2, 7]. While primary closure offers the advantage of less scar tissue and cosmetic benefits, it may not be suitable for cases involving large resections of the lip [7–9]. In cases where major defects of the lower lip need repair, different modalities such as free flap should be utilized because the defect cannot be closed primarily [9, 10].

Free flaps were first described in 1959, and since then, they have become the gold standard for functional and aesthetic reconstruction of large defects resulting from head and neck cancer tumor resections [11, 12]. Vascular anastomosis is a vital step in microvascular free flap surgeries, and for the procedure to succeed, close monitoring of the flap is necessary to avoid flap failure [13]. The most common etiologies for flap failure occur immediately after the operation, while the free flap depends entirely on the pedicle for vascularity. Improper microvascular anastomosis can lead to mechanical obstruction of the vascular pedicle of the flap and result in flap failure [14]. One of the situations facing almost all microvascular surgeons is when they cannot make up their minds clearly whether anastomosis works or not in the end. Two preoperative requirements are necessary for any microvascular surgeon, one is being familiar with different techniques of this type of surgery, and the other is having a deep knowledge of the vascular anatomy [15]. In this situation, most surgeons decide to repeat anastomosis, which takes longer, and there is even a higher chance of vascular and flap damage. Sometimes, they choose to keep anastomosis with further close observations, which has its risks. This paper reports the Y-shaped anastomosis, which is an end-to-end anastomosis between three vessels, practically adds the second end to the side anastomosis to a primary doubtful end-to-end connection instead of redoing the given procedure.

## Case report

**Fig. 1:**
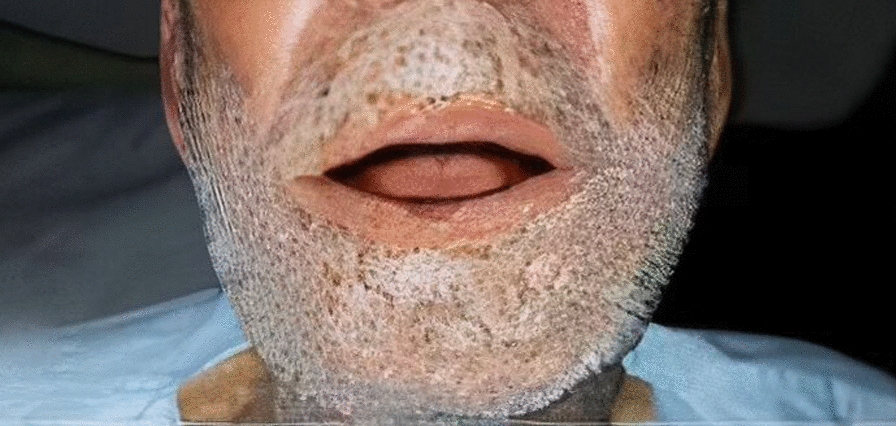
Before operation

**Fig. 2:**
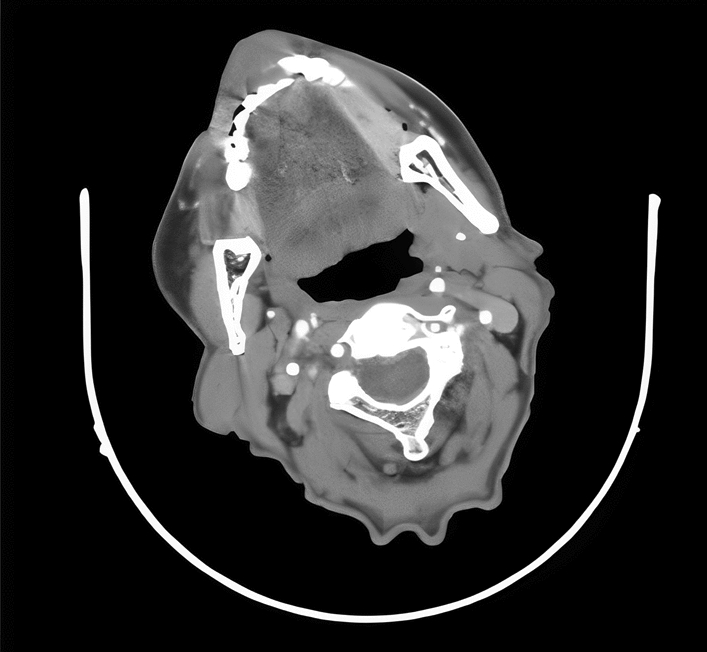
Preoperative CT scan

A 77-year-old Persian man with a 1.5 cm × 1 cm non-ulcerative lesion in the midline of the lower lip (Fig. 1) for 5 months, which had gradually increased in size, is reported. The lesion had some irregular edges without any satellite lesions. After examination, it was firm and painless with induration of the surrounding skin and mild inflammation. Neck examination also showed suspicious lymph nodes on the right side at level 2, as confirmed in the computerized tomography (CT) scan (Fig. 2). The examination of the rest of the oral cavity was normal.

The patient had worked as a farmer over the past 50 years, with a history of smoking (for 50 years), and was not an alcohol consumer. Besides, the biopsy had been taken around 3 months before the patient came to the private office and was reported as well-differentiated (SCC).

**Fig. 3:**
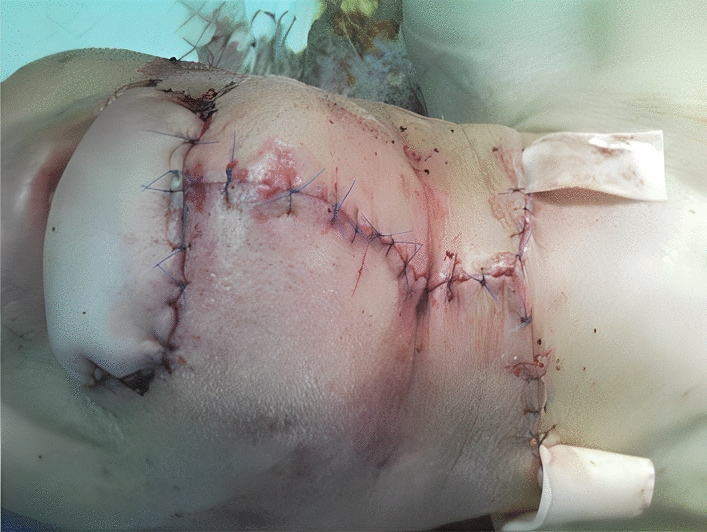
Immediately after the operation

**Fig. 4:**
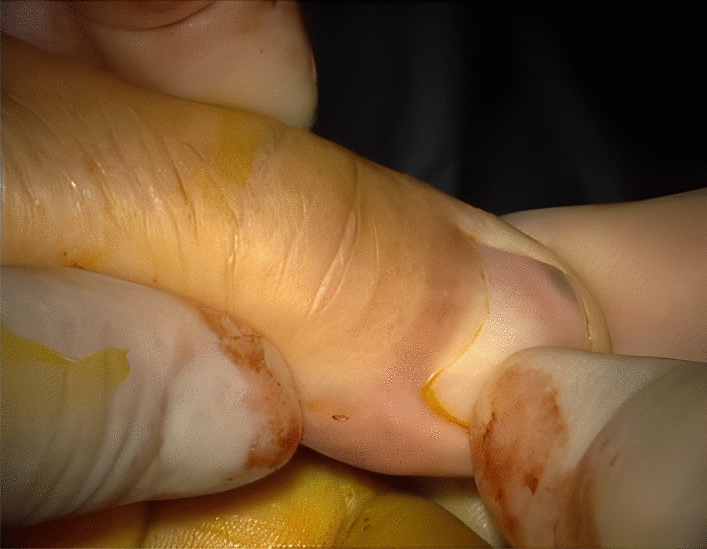
Allen test

**Fig. 5:**
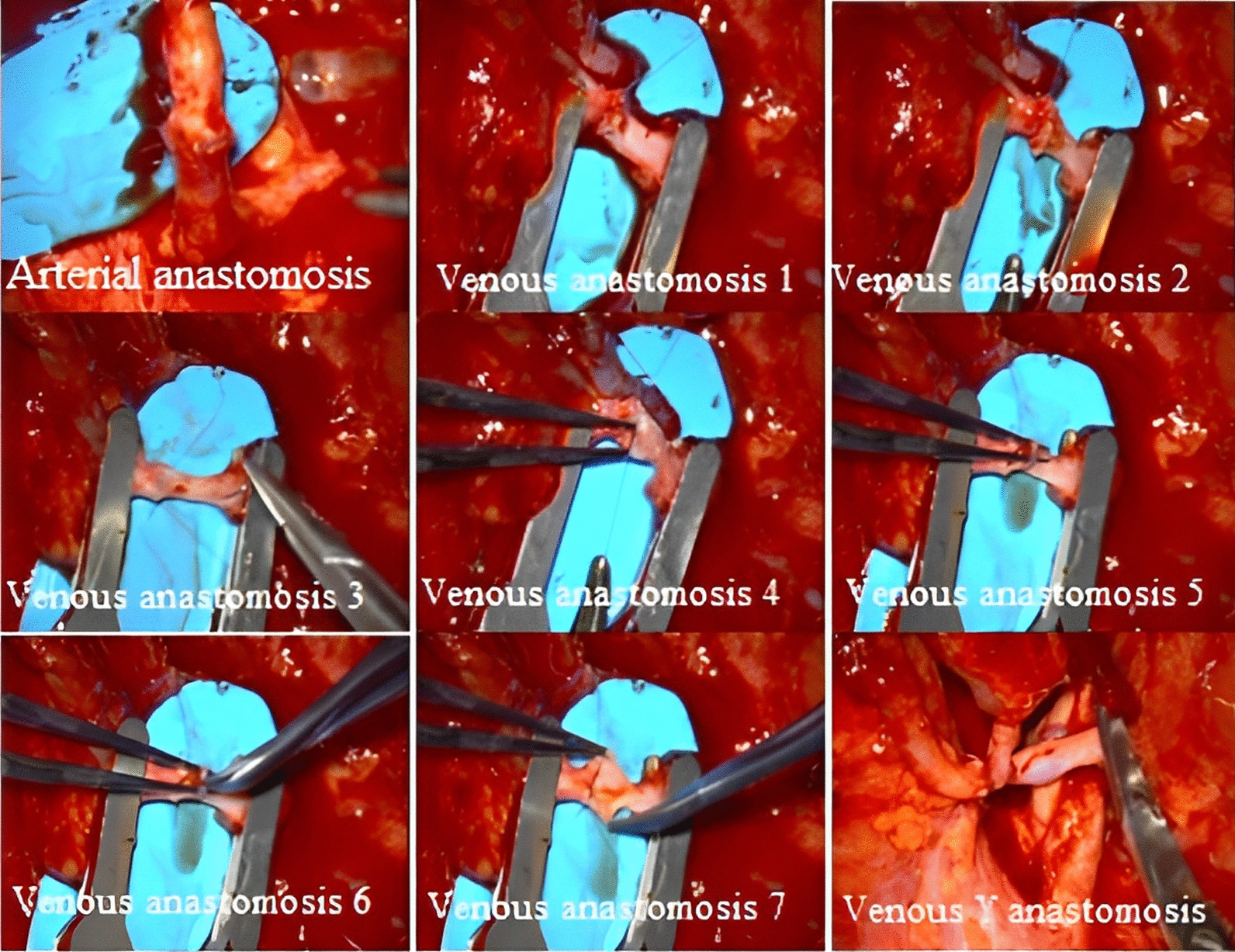
Step by step of anastomosis

**Fig. 6:**
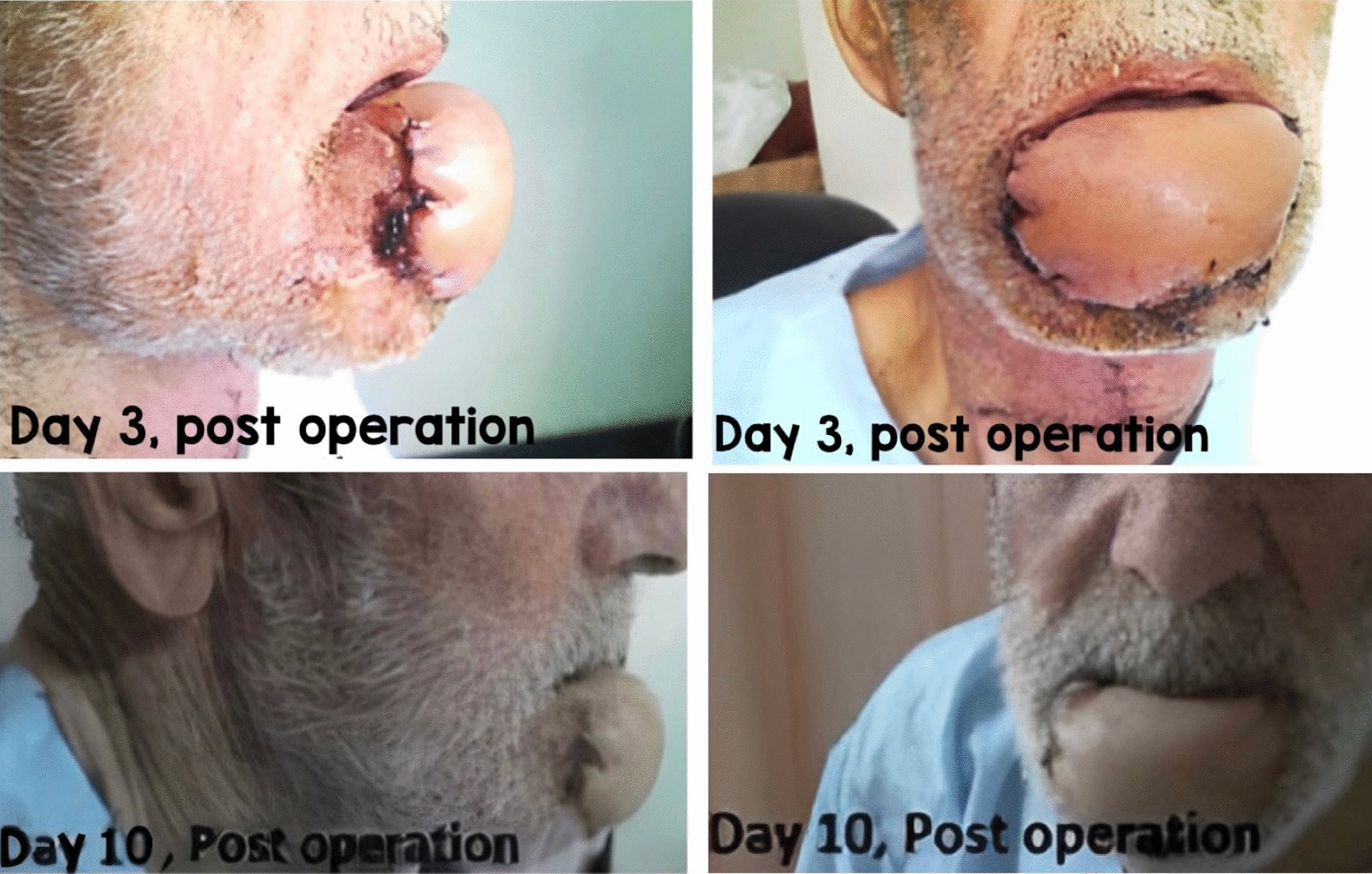
Follow-up

His treatment plan was the SCC resection with a 2 cm safety margin, the bilateral neck dissection (the right radical neck dissection and the supraomohyoid neck dissection on the left side) [16], and the lower lip reconstruction via the right radial forearm free flap (RFFF), known as the Chinese flap [17, 18] (Fig. 3) (of note, the case was right-hand dominant). The donor site was thus covered by a split-thickness skin graft, harvested from the right groin, and then closed primarily. The arterial pedicle of the free flap was also taken from the radial artery. The Allen test [19] was also performed before the surgery (Fig. 4), and the venous pedicle was taken from the cephalic vein. No nerve graft was taken. The arterial pedicle was subsequently anastomosed to the facial artery (end-to-end), and the venous pedicle was anastomosed to the facial vein (end-to-end), which was doubtful. Hence, the surgeon decided to add another end to the side anastomosis on the same pedicle instead of redoing the connection (Figs. 5, 6). The median follow-up time was 6 months to 1 year, and there was no locoregional recurrence. The flap has successfully survived during post-operative follow-up examinations. Notably, no related complications were observed, such as the need for re-exploration, venous thrombosis, hematoma, fistula, infection, or partial or total flap necrosis.

## Discussion

In the context of free-flap reconstruction microsurgery challenges, when confronted with uncertainty regarding the adequacy of vascular anastomosis, practitioners commonly have three primary options to consider and evaluate: redoing the anastomosis, adding a second anastomosis, or adopting a wait-and-watch approach.

Each modality comes with its own set of advantages and disadvantages; the redo strategy, although a potential solution, has its drawbacks [20]. It can be time-consuming, leading to fatigue for both surgeons and the medical team. Moreover, it increases the duration of surgery and anesthesia, and opening sutures may traumatize the vascular ends on both sides, which is less than ideal. Shortening and refreshing the vascular graft can also be problematic, especially when dealing with short pedicles [6, 20] The second option, involving the addition of a second anastomosis, is also time-consuming and not always feasible since surgeons typically prepare only one pedicle for each vein and artery, limiting the availability of a second viable option. The third strategy, the wait-and-watch approach, may lead to losing precious time in microvascular surgeries, potentially adding to the risk and emotional stress of a second surgery within a short period [20, 21].

However, Y anastomosis presents itself as a potential alternative with the advantage of mitigating many of the mentioned disadvantages, except for the time consumption aspect [22]. Nevertheless, it has its own set of drawbacks, including the risk of traumatizing the lateral wall of the receiving vessel shortly after the end-to-end anastomosis and the possibility of inducing turbulent flows, which could affect the normal linear blood flow [23, 24].

Miao *et al.* undertook a complex reconstructive head and neck surgery on a 44-year-old male patient with a tongue SCC recurrence. The patient had a medical history of undergoing modified radical neck dissection and salvage chemoradiotherapy. This prior treatment posed significant challenges during this surgery, particularly in selecting a suitable recipient vessel and dealing with the discrepancy in vessel diameters. The surgical team utilized an anterolateral thigh (ALT) free flap to address these difficulties for reconstruction. They employed the innovative II-Y-shaped technique for the crucial vascular anastomosis, known for its effectiveness in vessel-depleted areas. This technique proved to be an efficient way of adapting to the limited vessel diameters caused by previous surgeries and radiotherapy [25].

In a retrospective study of 98 patients, Scaglioni *et al.* compared the “Open-Y” technique with the conventional technique for free tissue transfer reconstruction. The “Open-Y” group showed a higher flap success rate (100% versus 96.5% in the conventional group) and fewer complications (10% versus 31% in the conventional group). Although the “Open-Y” group had a slightly higher size discrepancy rate, the results were significantly better overall [26].

Chen *et al.* also compared the “Open-Y” technique with the conventional technique for end-to-end anastomoses between the STA and donor arteries in 337 head and neck reconstruction patients. The “Open-Y” group had a high flap success rate of 98.6% (71/72), similar to the conventional group (97.4%; 245/252). However, the “Open-Y” group showed a significantly higher size discrepancy rate (66.7% versus 11.7% in the conventional group). There was no significant difference in arterial anastomotic site-related complications between the groups (1.4% versus 4.2%). Other complications had similar presentations in both groups [23].

In their study, Boeckx *et al.* thoroughly investigated the clinical applications of the Y-shaped anastomosis, explicitly concentrating on venous anastomoses in a diverse range of flap types, including the gracilis, latissimus dorsi, rectus abdominis, and others. Remarkably, out of the total 60 Y anastomoses performed, they achieved an impressive success rate of 95% [24].

Microvascular anastomosis requires careful consideration of factors like recipient area anatomy, flap pedicle anatomy, and flap flow volume. As clinical situations vary, there is no universal microvascular technique, necessitating surgeons to choose the most appropriate method for each case’s unique characteristics [24].

The Y-shaped anastomosis stands out as an innovative and versatile technique, offering comparable reliability and patency rates to conventional methods. Future research should encompass a larger number of cases to gain deeper insights into its effectiveness and potential complications, allowing for further refinements in its application in microvascular surgeries [24, 26].

## Conclusion

Microsurgical anastomosis of the vein and artery is a crucial aspect of free flap surgery, which is considered a gold standard in head and neck reconstructions. However, a common complication encountered during vascular anastomosis is the size mismatch between the vein and artery. The “Y” anastomosis approach proves to be a valuable solution to address this issue, making free-flap surgeries more manageable and effective.

## Data Availability

Not applicable.
